# Magnetically Propelled Microrobots toward Photosynthesis of Green Ammonia from Nitrates

**DOI:** 10.1002/smll.202407050

**Published:** 2024-11-11

**Authors:** Apabrita Mallick, Jeonghyo Kim, Martin Pumera

**Affiliations:** ^1^ Advanced Nanorobots & Multiscale Robotics Laboratory Faculty of Electrical Engineering and Computer Science VSB − Technical University of Ostrava 17. listopadu 2172/15 Ostrava 708 00 Czech Republic; ^2^ Department of Medical Research China Medical University Hospital China Medical University No. 91 Hsueh‐Shih Road Taichung 4040 Taiwan

**Keywords:** ammonia, magnetically driven, microrobots, nitrate reduction, photosynthesis

## Abstract

Ammonia (NH₃) production is a critical industrial process, as ammonia is a key component in fertilizers, essential for global agriculture and food production. However, the current method of synthesizing ammonia, the Haber‐Bosch process, is highly energy‐intensive, and relies on fossil fuels, contributing substantially to greenhouse gas emissions. Moreover, the centralized nature of the Haber‐Bosch process limits its accessibility in remote or resource‐limited areas. Photochemical synthesis of ammonia, provides an alternate lower energy, carbon‐free pathway compared to the prevailing industrial methods. The photoconversion of nitrate anions, often present in wastewater, offers a greener, more sustainable, and energy‐efficient route for both ammonia‐generation and wastewater treatment. Photochemical and chemical synthesis of ammonia requires intensive mass‐transfer processes, which limits the efficiency of the method. To change the game, in this work, a key new technology of ammonia‐generation, a catalytic ammonia generation (AmmoGen) microrobot, which converts nitrate to ammonia using renewable light energy is reported. The magnetic propulsion of the AmmoGen microrobots significantly enhances mass‐transfer, and expedites the photosynthesis of ammonia. Overall, this “proof‐of‐concept” study demonstrates that microrobots can aid in catalytic small molecule activation and generation of value‐added products; and are envisaged to pave the way toward new sustainable technologies for catalysis.

## Graphical abstract

Magnetically propelled ammonia‐generation (AmmoGen) microrobots are showcased in this work which can convert nitrates (NO₃^−^), a potential wastewater contaminant to value‐added green ammonia (NH₃) using renewable light energy, via a sustainable, low‐energy, and carbon‐free pathway. The magnetic propulsion significantly enhances the mass‐transfer toward and from the photocatalytic AmmoGen microrobots and facilitates the photosynthesis of ammonia.

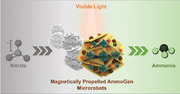

## 1 Introduction

Ammonia is an essential and versatile chemical because it acts as a feedstock to fertilizers, carbon‐free fuels, and fine chemicals.^[^ ^1^, ^2^, ^3^ ^]^ The current global production for ammonia is ≈170 million metric tonnes and it is majorly harnessed from the industrial Haber–Bosch process, which combines gaseous nitrogen (N_2_) and hydrogen (H_2_) to produce ammonia.^[^ ^4^, ^5^ ^]^ Nevertheless, this process is quite energy‐intensive as it requires high temperature (≈300–500 °C) and pressure (≈100–300 atm).^[^ ^6^, ^7^ ^]^ Further, the production of such high energy leaves behind a considerable carbon footprint (≈1–2% of global CO_2_ production per year).^[^ ^8^, ^9^, ^10^ ^]^ Hence, the quest begins for the production of green ammonia in a sustainable fashion under ambient reaction conditions. The use of clean and renewable energy sources like light and electricity has been sought over recent years to seek alternative routes to decarbonize the production of ammonia. In this pursuit, novel catalysts have been designed, extending from conventional iron (Fe)‐based catalysts^[^ ^11^, ^12^ ^]^ to cobalt (Co),^[^ ^13^, ^14^ ^]^ copper (Cu),^[^ ^15^, ^16^ ^]^ nickel (Ni),^[^ ^17^, ^18^ ^]^ manganese (Mn),^[^ ^3^, ^19^ ^]^ and ruthenium (Ru)^[^ ^20^, ^21^ ^]^‐based metallic or bimetallic catalysts, operating under milder reaction conditions.

The reduction of N‐containing species like dinitrogen or nitrates utilizing light or electrical energy sources is the most prevalent technique developed recently for producing ammonia. Typically, a nitrogen reduction reaction (N_2_RR) to ammonia requires 6 electrons, whereas a nitrate reduction reaction (NO_3_^−^RR) to ammonia requires 8 electrons, apparently giving an impression that N_2_RR is more energy‐efficient.^[^ ^22^ ^]^ Nonetheless, the nitrogen reduction reactions are perturbed by several limitations. From the standpoint of energy, nitrates are more sustainable N‐containing sources as the bond dissociation energy of the highly stable N≡N bond (941 kJ mol^−1^) in dinitrogen is much higher when compared with nitrates (204 kJ mol^−1^).^[^ ^23^ ^]^ Moreover, the low solubility of dinitrogen in water and its lesser affinity to protons also limits the usage of nitrogen reduction reactions to produce ammonia.^[^ ^24^ ^]^ On the contrary, nitrates are more soluble in water, have relatively lower N═O bond dissociation energy, and are quite abundantly found as pollutants in groundwater and industrial wastewater.^[^ ^25^ ^]^ Considering all these factors, the photoreduction of nitrates provides an alternative greener route toward sustainable ammonia synthesis. Furthermore, this process can simultaneously maintain the balance in the global nitrogen cycle and help in the purification of nitrate‐contaminated wastewater. In this light, the design of an efficient material is necessary which can boost the efficiency of nitrate reduction, and can in principle function more efficiently than conventional catalysts.

Micro/nanorobots are miniature, artificial, self‐propelling devices operating at materials science and nanotechnology interfaces.^[^ ^26^, ^27^ ^]^ Multiple propulsion techniques have been deployed to power these robots, encompassing chemical, enzyme, light, magnetism, electricity, and acoustics.^[^ ^28^, ^29^, ^30^, ^31^, ^32^, ^33^, ^34^, ^35^ ^]^ These robots have been significantly implemented over recent years for diverse applications like removing micro/nanoplastics,^[^ ^36^, ^37^ ^]^ eradicating biofilm,^[^ ^38^ ^]^ sensing,^[^ ^39^ ^]^ and therapeutics.^[^ ^40^, ^41^ ^]^ For catalytic reactions, magnetic propulsion of the catalytic microrobots is a very promising methodology as it offers facilities like easy separation and retrieval from the reaction medium, and waives off the usage of any external chemicals that might lead to contamination of the reaction medium and perturbation of the catalytic reaction. The motion of the magnetic robots is controlled only by the application of an external rotating magnetic field and this magnetic field does not hinder the chemical reaction. Additionally, the magnetic propulsion also induces fluid mixing and increases the interaction between the catalytic microrobots and the substrates; thereby, enhancing the catalytic processes. However, though different magnetic microbots have been used to date for photocatalytic reactions like degradation of toxic chemicals like nerve agents^[^ ^42^ ^]^ and sunscreens,^[^ ^43^ ^]^ and thus help in wastewater purification; to the best of our knowledge, magnetic robots have not been explored much till now for the production of green energy.^[^ ^44^ ^]^

**Figure 1:**
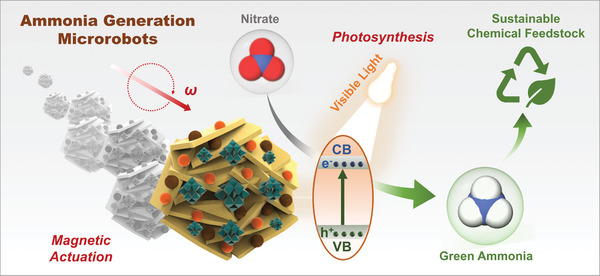
Schematic representation of magnetically propelled AmmoGen microrobots producing green ammonia from nitrates under visible light irradiation.

**Figure 2:**
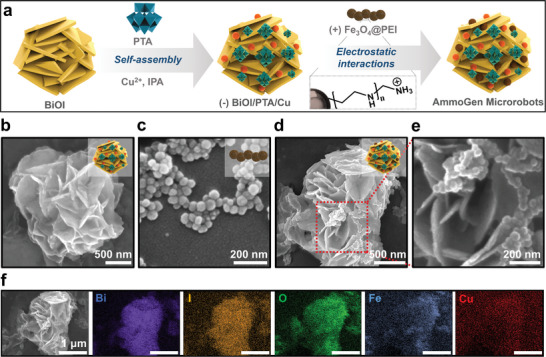
Fabrication of the AmmoGen microrobots. a) Schematic representation of the fabrication of the AmmoGen microrobots. Scanning Electron Microscopy (SEM) images of b) BiOI/PTA/Cu microparticles. c) Fe_3_O_4_@PEI particles. d) AmmoGen microrobots formed by electrostatic interactions between (b,c). e) Magnified image of the surface of (d) showing spherical Fe_3_O_4_@PEI nanoparticles deposited on the surface. f) Energy Dispersive X‐Ray (EDS) mapping of the elements present in the AmmoGen microrobot.

In this work, we demonstrate magnetic ammonia‐generation (AmmoGen) microrobots for the photosynthesis of ammonia from nitrates as illustrated in **Figure** **1**. The fabrication of the desired microrobot for the photosynthesis of ammonia involves two components, that is, the photoactive counterpart for the catalysis which is composed of a semiconductor‐based microrobot decorated with phosphotungstic acid and copper (BiOI/PTA/Cu); and the magnetic counterpart for actuation which comprises iron oxide functionalized with terminal amine; assembled via the electrostatic interactions between the two counterparts (**Figure** **2**a). The magnetic component of the AmmoGen microrobots ensures their maneuvering in a controlled fashion by the rotating magnetic field. The hybridized photocatalytic part reduces the nitrate to value‐added ammonia under ambient conditions in the presence of visible light. The magnetic actuation of the microrobots can enhance the photocatalytic generation of ammonia owing to their self‐propulsion and increased interaction between the catalysts and the nitrates. Moreover, these microrobots can be easily separated and retrieved from the catalytic mixture without complex purification technologies. This work, in principle, can open up alternative, more efficient, and sustainable low‐carbon routes toward ammonia photosynthesis.

## 2 Results and Discussion

### 2.1 Fabrication and Characterization of the Ammogen Microrobots

The AmmoGen microrobots are devised using photoactive and magnetic hybrid materials and the schematic procedure for fabrication is illustrated in Figure 2a. Initially, photoactive bismuth oxyiodide and BiOI particles were synthesized in an aqueous phase following the technique described in the literature.^[^ ^43^ ^]^ The flower‐shaped BiOI particles were then functionalized with phosphotungstic acid polyoxometalate, H_3_[PW_12_O_40_], represented as PTA to form the BiOI/PTA composites. Subsequently, the BiOI/PTA composites were exposed to UV light irradiation for 3 hours, under an N_2_ environment and in the presence of isopropanol. Polyoxometalates have excellent redox properties^[^ ^45^, ^46^ ^]^ and under these reaction conditions, the UV‐light switchable {PW_12_O_40_}^3^^−^ moieties bound to BiOI were reduced to {PW_12_O_40_}^4^^−^, represented as PTA*.^[^ ^47^, ^48^ ^]^ In the next step, Cu^2+^ ions were introduced into the BiOI/PTA^*^ dispersion in the form of CuCl_2_.2H_2_O and the dispersion was allowed to mature for 12 h. The bound PTA^*^ moieties on the BiOI surface function as the highly localized reducing agents for reducing metallic Cu^2+^. With time, PTA* was reversibly oxidized back to PTA and simultaneously Cu^2+^ was reduced to Cu^+^/Cu^0^, in the presence of isopropanol. The reactions can be represented by the following Equations (1) and (2).^[^ ^47^ ^]^

(1) $${\left\{PW_{12}O_{40}\right\}}^{3-}+{\left(CH_{3}\right)}_{2}\mathrm{CHOH}\to {\left\{PW_{12}O_{40}\right\}}^{4-}+{\left(CH_{3}\right)}_{2}\mathrm{CO}$$

(2) $${\left\{PW_{12}O_{40}\right\}}^{4-}+Cu^{2+}\to {\left\{PW_{12}O_{40}\right\}}^{3-}+Cu^{+}/Cu^{0}$$

**Figure 3:**
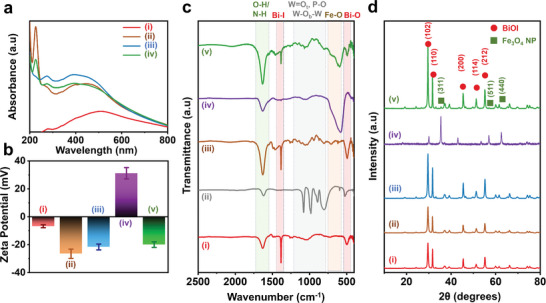
Characterization of the AmmoGen microrobots. a) UV–vis spectroscopic studies of the materials synthesized step‐by‐step: i) BiOI, ii) BiOI/PTA, iii) BiOI/PTA/Cu, and iv) AmmoGen microrobots; b) Zeta potentials of the materials synthesized step‐by‐step: i) BiOI, ii) BiOI/PTA, iii) BiOI/PTA/Cu, iv) Fe_3_O_4_@PEI, and v) AmmoGen microrobots. Error bars indicate standard deviations from three replicate measurements; c) Powder X‐ray diffraction (PXRD) studies of i) BiOI, ii) PTA, iii) BiOI/PTA, iv) Fe_3_O_4_@PEI, and v) AmmoGen microrobots; D) Fourier‐transform infrared (FT‐IR) spectroscopic studies of i) BiOI, ii) BiOI/PTA, iii) BiOI/PTA/Cu, iv) Fe_3_O_4_@PEI, and v) AmmoGen microrobots.

The morphology of the as‐synthesized BiOI/PTA/Cu was monitored using scanning electron microscopy (SEM). SEM image in Figure 2b reveals the 3D flower‐shaped structure of the micrometer‐sized BiOI/PTA/Cu particles. The elemental mapping from energy‐dispersive X‐ray (EDS) measurements proved the presence of Bi, O, I, W, and Cu (Figure S1, Supporting Information). BiOI is a well‐known photocatalyst that shows photoactivity in the visible light region. Modification of BiOI with PTA and Cu reduces the recombination of electron–hole pairs, prolonging the lifetime of charge carriers, and thereby enhancing the photoactivity of the hybridized BiOI/PTA/Cu particles. The electron absorption spectrum (**Figure** **3**a) of BiOI/PTA/Cu shows a broad absorption profile in the visible light region, affirming that it is photoresponsive toward visible light (380–700 nm). The zeta (*ζ*) potential studies (Figure 3b) reveal that the BiOI/PTA/Cu composite has a negative surface charge of −22 ± 1 mV, owing to the presence of negatively charged polyoxometalate in the composite. This negatively charged BiOI/PTA/Cu composite forms the photoactive part of the microrobot for nitrate reduction to ammonia.

In the next step, to induce motility in the presence of a magnetic field, the negatively charged BiOI/PTA/Cu particles were further coated with spherical Fe_3_O_4_ nanoparticles (NPs). The Fe_3_O_4_ particles were synthesized via hydrothermal technique and were modified with a positively charged polyethylene imine (PEI) with terminal amide groups (Figure 2a). The opposite surface charges on the positively charged Fe_3_O_4_@PEI (*ζ *= +31 ± 1 mV) and negatively charged BiOI/PTA/Cu (*ζ* = −22 ± 1 mV) induced electrostatic interactions between them and thus the components were hybridized to form the AmmoGen microrobots (Figure 3b). Thereafter, the hybridized AmmoGen microparticles exhibited an intermediate surface charge of *ζ* = −19 ± 0.9 mV, proving stable electrostatic attachment of the BiOI/PTA/Cu and Fe_3_O_4_ NPs.

The value of Zeta potential for the microrobot is negative because the optimized loading of the positively charged Fe_3_O_4_ is low (experimental ratio of 1:10 by weight) when compared with the negatively charged BiOI/PTA/Cu. The morphology of the Fe_3_O_4_ NPs and the AmmoGen microrobot was further monitored using SEM. The SEM images in Figure 2c reveal that the Fe_3_O_4_ NPs are spherical with an average diameter of 50 ± 10 nm. Post‐modification with BiOI/PTA/Cu, a few of these spherical Fe_3_O_4_ NPs remain randomly scattered on the surface of the flower‐like BiOI/PTA/Cu (Figure 2d,e). The loading of the Fe_3_O_4_ NPs was optimized in such a way that the magnetic particles are sufficient to impart motion to the microrobots, while on the other hand, most of the surface of the AmmoGen robots remains free for the surface reaction, which is essential for the reduction of nitrates. The elemental mapping from the EDS studies now manifests the presence of the element Fe in addition to the elements existing in BiOI/PTA/Cu (Figure 2f). The light absorption spectrum of the AmmoGen microrobots shows a similar broad absorption in the visible light region of the spectrum (Figure 3a) indicating that the photoactivity of the BiOI/PTA/Cu is preserved even after modification with magnetic Fe_3_O_4_ particles. The X‐ray diffraction (XRD) patterns also confirmed the formation of the AmmoGen microrobots (Figure 3c). From the acquired XRD pattern, the characteristic peaks of BiOI were observed at 2*θ* values of 9.6°, 29.6°, 31.8°, 45.4°, 51.2°, and 55.2° corresponding to planes (001), (102), (110), (200), (114) and (212) respectively (JCPDS number: 10–0445).^[^ ^43^ ^]^ The synthesized Fe_3_O_4_ NPs are crystalline with distinct peaks at 30.0°, 35.3°, 43.0°, 53.4°, 56.9°, and 62.5° which are attributed to the (220), (311), (400), (422), (511), and (440) planes respectively of the cubic phase of the lattice (ICCD 19–0629).^[^ ^49^ ^]^ After modification of BiOI/PTA/Cu with crystalline Fe_3_O_4_ NPs, additional peaks appeared at 2*θ* values at 35.3°, 56.9°, and 62.4° which might be assigned to the (311), (511), and (440) planes respectively for the cubic phase of Fe_3_O_4_; the peaks corresponding to the other planes are believed to be masked by the more intense peaks originating from BiOI. The formation of the AmmoGen microrobots was also analyzed using Fourier‐transform infrared (FT‐IR) spectroscopy. From the spectrum (Figure 3d), several peaks appear; the peaks at 498 and 1382 cm^−1^ correspond to the Bi─O and Bi─I interactions present in BiOI.^[^ ^50^ ^]^ The peaks at 571 and 1631 cm^−1^ arise from the Fe─O vibrations and N─H stretch/O─H bend respectively in Fe_3_O_4_@PEI counterparts.^[^ ^49^ ^]^ The small peaks in the range between 1100–800 cm^−1^ correspond to the W═O_t_, W─O_b_─W, and P─O bonds present in PTA molecules.^[^ ^51^ ^]^ The simultaneous presence of all these peaks in the FT‐IR spectrum indeed confirms the formation of AmmoGen hybrid microrobots.

**Figure 4:**
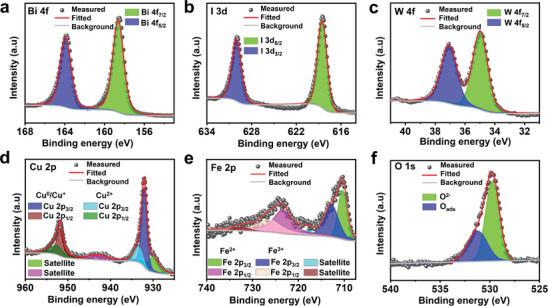
High‐resolution X‐ray photoelectron spectra (XPS) of the AmmoGen microrobots. Deconvoluted XPS of AmmoGen microrobots for core levels a) Bi 4f, b) I 3d, c) W 4f, d) Cu 2p, e) Fe 2p, and f) O 1s.

X‐ray photoelectron spectroscopy (XPS) was used to investigate the surface composition and chemical state of the microrobots. From the survey spectra (Figure S2, Supporting Information), the presence of the constituent elements, bismuth (Bi), iodine (I), tungsten (W), copper (Cu), iron (Fe), and oxygen (O) was confirmed. High‐resolution spectra were recorded for all the individual elements, followed by fitting the peaks of the spectra (**Figure** **4**), and the binding energy values for all the fitted peaks are also provided (Table S1, Supporting Information). The high‐resolution spectra of Bi 4f (Figure 4a) show two peaks at 158.6 and 163.9 eV binding energies corresponding to Bi 4f_7/2_ and Bi 4f_5/2_ of Bi^3+,^ while that of I 3d (Figure 4b) shows two peaks at 618.5 and 630.0 eV binding energies corresponding to I 3d_5/2_ and I 3d_3/2_ of I^−^ respectively, proving the presence of BiOI in the microrobot.^[^ ^43^ ^]^ From the high‐resolution W 4f spectra (Figure 4c), two peaks at 35.0 and 37.1 eV corresponding to W 4f_7/2_ and W 4f_5/2_ indicate the presence of phosphotungstic acid in the system.^[^ ^35^, ^52^ ^]^ However, Cu exhibits mixed valent states as evident from the deconvoluted Cu 2p core spectra (Figure 4d). The major intensity peaks at 931.9 and 951.8 eV arise due to Cu 2p_3/2_ and Cu 2p_1/2_ in Cu^+^/Cu^0^ states, the minor intensity peaks at 933.4 and 952.8 eV correspond to Cu^2+^, while two minimal satellite peaks also appear, which might be due to the formation of copper oxides.^[^ ^53^ ^]^ The deconvolution of the Fe 2p core‐level spectra also shows complex multiplet splitting (Figure 4e) in accordance with the literature. The major peaks appear at 710.3 and 723.6 eV due to Fe 2p_3/2_ and Fe 2p_1/2_ of Fe^2+^ in Fe_3_O_4_, the smaller and satellite peaks appear due to the presence of minor Fe^3+^.^[^ ^54^ ^]^ The O 1s core‐level spectra (Figure 4f) show two major peaks‐ the first one at 529.7 eV is assigned to the O^2−^ of oxides and the second one at 531.2 eV due to adsorbed oxygen atoms.^[^ ^55^ ^]^ After the fabrication and characterization of AmmoGen microrobots, they were subjected to a rotating magnetic field to observe their dynamic characteristics and this magnetic motion will be discussed in the subsequent section.

### 2.2 Magnetic Motion of the AmmoGen Microrobots

In this study, the AmmoGen microrobots comprise photoactive and magnetic components. The magnetic part is composed of Fe_3_O_4_ NPs coated with polyethylene imine with terminal amide groups and this magnetic component guides the magnetic motion of these microrobots. To induce motion in these microrobots, they were subject to a transversal rotating magnetic field using a customized six‐coil setup with tuneable magnetic field intensity and frequencies as described in the Experimental Section. Without the magnetic field, the particles only display Brownian motion in aqueous dispersions. When the magnetic field is applied, the Ammogen microrobots move along the *x–z* plane of the rotating magnetic field with a tumbling motion at a fixed angle, as is manifested in Movie S1 (Supporting Information), at 5 mT and 5 Hz frequency of the magnetic field.

**Figure 5:**
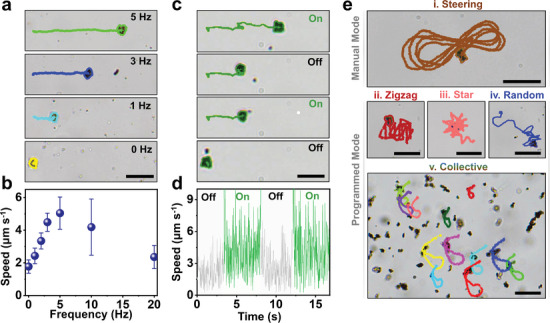
Magnetic propulsion of the AmmoGen microrobots. a) Propulsion trajectories of a single Ammogen microrobot represented by colored lines at different frequencies (*f* = 0, 1, 3, and 5 Hz) of the rotating magnetic field, Scale bar: 10 µm; b) Average instantaneous speed (mean ± standard deviation obtained for 20 individual particles) of the AmmoGen microrobots as a function of frequencies of the magnetic field; c) Propulsion trajectories of a single Ammogen microrobot represented by greenish lines by switching “on” and “off” the magnetic field; d) The speed of AmmoGen microrobots by switching “on” and “off” the rotating magnetic field. Gray and green points indicate the “off” and “on” conditions of the rotating magnetic field respectively; e) Coloured lines representing trajectories of the AmmoGen microrobots in automated and manual steering modes: i) Manual steering, ii) Zigzag mode, iii) Star‐shaped mode, iv) Random mode, and v) Collective motion. Scale bar: 10 µm.

To probe the magnetic actuation further, a single AmmoGen microrobot particle was monitored at different frequencies (*f*). **Figure** **5**a and Movie S2 (Supporting Information) illustrate the time‐lapse images and trajectories of the motile microrobot respectively at different frequencies (*f*) ranging from 0 to 5 Hz, while the magnetic field intensity is maintained at 5 mT. Under all of these experimental conditions, the AmmoGen microrobot shows linear propulsion with a tumbling rotation, which is synchronized with the rotational frequency in each case. The instantaneous propulsion speed also increases linearly along with the increasing frequency and this trend continues up to a maximum *f *= 5 Hz where the maximum instantaneous speed, 5.04 ± 0.98 µm s^−1^ is achieved for the AmmoGen microrobot. After *f* = 5 Hz, the motion of the microrobots becomes unsynchronized with the input frequencies, hence the frequency 5 Hz is termed as the “step‐out” frequency, and at higher frequencies of 10 and 20 Hz, it was observed that the instantaneous speed of the microrobots starts to decrease (Figure 5b). The reason for this unsynchronized motion at higher frequencies is that the viscous torque originating from the surrounding liquids overcomes the magnetic torque and diminishes the rotational as well as the linear propulsion speed of the microrobots.^[^ ^56^ ^]^ The magnetic motion of the AmmoGen microrobots can also be manipulated by switching on/off the magnetic field, as seen in Movie S3 (Supporting Information). The variation of the instantaneous speed versus time for the “on” and “off” states of the magnetic field is illustrated in Figure 5c,d. In the absence of the magnetic field, that is, in the “off” state, the microrobots solely exhibit Brownian motion, and in the presence of the magnetic field, that is, in the “on” state, they manifest directional motion with increased value of instantaneous speed, with respect to time. All these results reflect the fact that the magnetic actuation of the AmmoGen microrobots is spontaneous and their motion can be entirely controlled by an external rotating magnetic field.

Additionally, the motion of these AmmoGen microrobots can be controlled by switching the rotation angle of the magnetic field, which allows precise navigation. In this work, we show the proof of full spatial control of these microrobots using “manual” as well as “programmed” steering. In Figure 5e, the trajectories of the propulsion of the microrobots in both “manual” and different “programmed” modes have been demonstrated. In “manual” navigation mode, the rotation angle of the magnetic field is adjusted manually such that the microrobot is steered along a projected “infinite” shaped trajectory (Figure 5e–i, Movie S4, Supporting Information). For programmed steering, a magnetic controller is employed which can alter the rotation angles (*α*_i_) with respect to time and can generate predefined navigation modes like “zigzag” (Figure 5e‐ii), “star‐shaped” (Figure 5e‐iii), or “random” (Figure 5e‐iv) motions of the microrobots in repeated cycles (Movie S5, Supporting Information). Swarming effects for a collection of these particles were also monitored using the “random” mode of propulsion (Figure 5e‐v, Movie S6, Supporting Information). For all these experiments, the magnetic field was maintained at *f *= 5 Hz and *B* = 5 mT. In “random” mode, the magnetic controller randomly changes the rotation angle between 0° ≤ *α*_i_ ≤ 360° every alternate second, which induces automated perpetual motion in the AmmoGen microrobots, promoting local fluid convection and magnetic self‐stirring.^[^ ^56^, ^57^, ^58^ ^]^ Hence, this programmed random mode can be utilized to stimulate various processes, like photochemical reactions,^[^ ^42^ ^]^ biofilm eradication,^[^ ^31^, ^38^ ^]^ water cleaning,^[^ ^59^ ^]^ and the capture of micro/nano plastics.^[^ ^36^, ^37^ ^]^ In this work, we apply this programmed random mode for steering the AmmoGen microrobots, and these microrobots can promote the production of ammonia from nitrates, which will be discussed in the next section.

### 2.3 Photosynthesis of Green Ammonia by the Magnetically Propelled AmmoGen Microrobots

Ammonia is the key starting material for the industrial production of nitrogen‐containing chemicals and, hence its production under ambient conditions is essential in current times. Ammonia production by photocatalysis using visible light irradiation is an emergent field of research because it can harness renewable energy sources, and is a sustainable approach to generate valuable ammonia from nitrates. Nitrates are abundantly found as contaminants in industrial wastewater as well as groundwater.^[^ ^25^ ^]^ The conventional photocatalytic production of ammonia involves the interaction of the precursor nitrates with the catalysts via adsorption, followed by its reduction to produce ammonia. Microrobots with their autonomous propulsion technology offer unique characteristics like precise control of motion, enhanced interactions between molecules and microrobots, and local fluid convection. Previously, different light‐driven and magnetically propelled microrobots have been used for wastewater treatment via photocatalytic degradation of divergent pollutants like nerve agents, sunscreen, herbicides, and nitroaromatic compounds.^[^ ^60^ ^]^ Banking on all these characteristics of the microrobots, in this work we fabricate an AmmoGen microrobot for producing ammonia from nitrates. The AmmoGen microrobot is designed in such a way that it has the components for both photocatalytic activity and magnetic propulsion. The photocatalytic part photoreduces the nitrates powered by a visible light source to generate ammonia, and simultaneously the magnetic particles impart magnetic actuation to the microrobots which aid in the photocatalytic reaction. Magnetic propulsion also eliminates the need for externally added fuels like H_2_O_2_ which has strong redox properties and might impede the nitrate reduction reactions.

Photocatalytic nitrate reduction to ammonia in acidic medium generally involves 8e^−^ reduction of the nitrate and proceeds via the following reaction:^[^ ^14^ ^]^

(3) $${{\mathrm{NO}}_{3}}^{-}+9H^{+}+8e^{-}\to {\mathrm{NH}}_{3}+3H_{2}O$$

**Figure 6:**
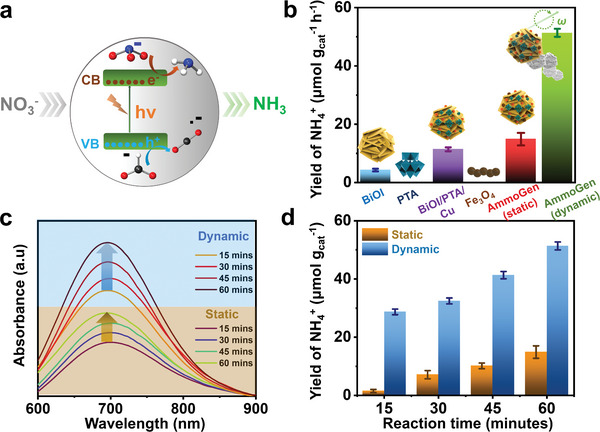
Ammonia production by the AmmoGen microrobots. a) Schematic representation of the AmmoGen microrobots producing ammonia from nitrates; b) Comparison of the yields of ammonia generated by the different individual constituents and the “static” and “dynamic” AmmoGen particles; c) UV–vis spectroscopic measurements showing the production of ammonia by the indophenol blue method. The spectra in the brown and blue zones correspond to the ammonia produced by “static” and “dynamic” particles respectively for aliquots drawn at time intervals of 15, 30, 45, and 60 min; d) Comparison of the ammonia yields by the “static” and “dynamic” AmmoGen microrobots at different reaction time intervals of 15, 30, 45, and 60 min. Error bars correspond to the standard deviation of data obtained from three replicate experiments.

Semiconductors like TiO_2_, WO_3_, and g‐C_3_N_4_, along with their modified structural hybridized materials, and donor–acceptor polymers have been used previously for photocatalytically reducing nitrates to ammonia.^[^ ^23^, ^61^, ^62^, ^63^ ^]^ In this work, a BiOI semiconductor hybridized with H_3_[PW_12_O_40_] polyoxometalate, Cu, and Fe_3_O_4_ forms the AmmoGen microrobot. Upon visible light irradiation, BiOI absorbs photons which excites the electrons to the conduction band and holes are simultaneously generated in the valence band (**Figure** **6**a). The polyoxometalate has W in +VI oxidation state and W^VI^ can accept the electrons from the conduction band of BiOI to form W^V^ and thus help in reducing the rate of electron–hole recombination.^[^ ^47^, ^48^ ^]^ Copper, an inexpensive, transition metal compared to noble metals; also has unique features like efficient charge separation and shows prominent activity for photo/electrochemical ammonia generation.^[^ ^64^, ^65^ ^]^ Hence, the hybridization of BiOI with polyoxometalate and copper enhances the photocatalytic activity of the microrobot. The reaction medium contains nitrate and formic acid. Formic acid maintains the pH of the reaction medium and acts as a hole scavenger. The photoreduction mechanism is illustrated in Figure 6a. The photogenerated holes (h_VB_^+^) formed in the valence band oxidize formate to carboxyl radical anion. Simultaneously, the photoexcited electrons in the conduction band (e_CB_^−^) reduce the nitrates (NO_3_^−^) to produce ammonia (NH_3_) as demonstrated in Equation (3). The electron absorption studies (Figure 3a) suggest that the designed AmmGen microrobot is photoactive in the visible region of the spectrum. The efficiency of a photocatalytic reaction depends on the different reaction parameters, so to perform a reaction systematically, optimization of the reaction parameters is essential. Initially, to optimize the reaction conditions, the photocatalysis was performed in different light sources‐ full spectrum, blue LED, and green LED illuminations (experimental details), and the yield of ammonia was found to be the maximum in green light (Figure S3, Supporting Information). Hence, all subsequent experiments hereafter, have been conducted in green light. Furthermore, to increase the efficiency of the photoconversion of nitrates to ammonia, the reactions were tested in the presence of different sacrificial agents.^[^ ^23^, ^66^ ^]^ To optimize the sacrificial agent, trial reactions were performed by adding phenol, ethylene glycol, benzyl alcohol, and formic acid (Figure S4, Supporting Information) to the reaction mixtures. The yield of ammonia was observed to be maximum in the case of formic acid for the photocatalytic experiments with the “static” as well as “dynamic” AmmoGen microrobots, hence formic acid was used in all of the forthcoming experiments. Control experiments were performed with the components of the microrobots, from which it has been evident that, though BiOI itself can produce a small amount of ammonia, the production of ammonia increases with the incorporation of {PW_12_O_40_} and Cu (Figure 6b). Additionally, control experiments were also performed under three reaction conditions (without light, without nitrate feedstock, and without AmmoGen microrobots) to validate the formation of ammonia by photoactivation of the AmmoGen microrobot from nitrates (Figure S5, Supporting Information). In the next set of planned experiments, we attempt to probe the impact of magnetic motion on the photocatalytic production of ammonia by the AmmoGen microrobots.

With this objective, the kinetic study of ammonia generation from nitrates by the AmmoGen microrobots was performed under visible light irradiation (green LED, *λ*_max _= 523 nm). Initially, a “dynamic” magnetic assay was prepared by dispersing the AmmoGen microrobots in an aqueous solution of nitrates containing formic acid as the hole scavenger. The dispersion was deaerated with purging Argon gas. This “dynamic” assay was subject to a transversal rotating magnetic field (5 Hz, 5 mT) under a “programmed” mode of motion and was illuminated with green light vertically from the top of the magnetic setup for 1 h. During the reaction, aliquots were drawn at regular intervals of time (*t*) = 15, 30, 45, and 60 min to evaluate the production of ammonia. The yield of ammonia produced was quantified by the indophenol blue method from the calibration curve obtained by UV–vis spectroscopy (Figure S6, Supporting Information).

To evaluate the promotional effect of magnetic motion in boosting the production of ammonia, a “static” assay was prepared with the AmmoGen particles. All the experimental conditions were kept unaltered, except implementing the transversal rotating magnetic field. In this case, too, aliquots were drawn at similar time intervals and were subject to an indophenol blue test followed by UV‐spectroscopic studies. The comparative studies between the “static” and “dynamic” assays are illustrated in Figure 6c,d. The absorbance at *λ*_max_ corresponding to indophenol blue is more in the case of the “dynamic” assay (indicated by blue zone) when compared with the “static” assay (indicated by brown zone) in Figure 6c. The yield of ammonia by the AmmoGen microrobots in “dynamic” mode increases up to 51.3 µmol g_cat _h^−1^, after one hour of reaction, whereas, the yield by the “static” AmmoGen particles is only 14.9 µmol g_cat _h^−1^. The yield of ammonia is monitored after 15, 30, 45, and 60 min of the reaction for the “static” and “dynamic” AmmoGen particles respectively. For each set, the produced ammonia is observed to be more in the case of the “dynamic” particles (Figure 6d). The reason for the increased yield of ammonia can be attributed to the increased interaction between nitrates and the catalytic AmmoGen microrobots, “in situ” cleaning of the surface of the microrobots, thus making the active sites present on the surface of the catalyst more accessible for photocatalytic reactions. Moreover, the magnetic propulsion of the AmmoGen microrobots imparts mass‐transfer which significantly impacts and enhances the yield of ammonia. Additionally, the magnetic AmmoGen microrobots can be easily recovered from the reaction medium using a simple neodymium magnet. To gain more insight into the effects of magnetic field strengths on the yields of ammonia, the photocatalytic reactions were carried out at frequencies of 0 Hz (“static”), 1 Hz, and 5 Hz (Figure S7, Supporting Information). It has been observed that the “dynamic” robots yield more ammonia than the “static” particles (0 Hz). Nevertheless, it has also been noted that the yields of ammonia obtained at 1 and 5 Hz frequencies are almost comparable. The exact impact of the magnetic field on photocatalysts includes changes in spin states, local electronic structures that impact charge transfer, and photoreactivity that require a series of complex, in‐depth studies to acquire more mechanistic insights in future works.^[^ ^67^ ^]^ In this work, we demonstrate an archetype showcasing how microrobots can aid the activation of small molecules like nitrate and generate value‐added ammonia. Previously published literature reports indicate that mechanical stirred and magnetically actuated microrobots have similar performance and efficiency, but magnetic propulsion of microrobots provides additional benefits including more precise control and more flexible reaction conditions than mechanical stirring.^[^ ^68^, ^69^ ^]^ With the aid of magnetic microrobots, the nitrate‐to‐ammonia photoconversion reactions can in principle be carried out in a small droplet, to a large industrial‐scale reaction vessel, to reactions in a confined space, where the use of mechanical stirrers like magnetic bars, renders moot.^[^ ^70^, ^71^ ^]^ Hence, this work can act as a proof of concept that the programmable magnetic maneuvering of the microrobots can efficiently photoreduce nitrates to produce more yield of valuable ammonia feedstock than conventional non‐motile photocatalysts, and can in principle pave up methods to design effective photocatalysts for small molecule activation reactions for generating value‐added chemicals.

## 3 Conclusion

In summary, this study presents the development and application of magnetically propelled AmmoGen microrobots for the photosynthesis of green ammonia from nitrates. The AmmoGen microrobots, composed of photoactive BiOI semiconductors hybridized with H_3_[PW_12_O_40_] polyoxometalate and Cu and decorated with Fe_3_O_4_ nanoparticles, demonstrate a significant enhancement in the mass‐transfer process through magnetic propulsion. This innovative approach leverages a rotating magnetic field to actuate the microrobots, thereby amplifying the catalytic interactions and boosting the ammonia yield by ≈3.4 times compared to “static” photocatalytic particles.

The results validate the effectiveness of magnetically driven microrobots in facilitating catalytic small molecule activation and the generation of value‐added products. The dynamic motion induced by the magnetic field enhances the interaction between the catalytic sites and nitrates, promoting a more efficient and sustainable ammonia production process. Moreover, these microrobots can be easily separated and retrieved from the reaction medium, simplifying the overall process.

This proof‐of‐concept study explores the prospects of magnetically propelled microrobots in catalytic applications, opening new avenues for developing advanced photocatalysts for green energy production. The findings pave the way for future research and development of sustainable technologies in catalysis, particularly for small‐molecule activation reactions like N_2,_ CO_2_, and NO_x_ reduction toward generating ammonia, methanol, ethanol, and other green fuels. The produced ammonia can act as storage for H_2_, eliminating the storage and transportation challenges associated with H_2_, thus promoting the net‐zero carbon‐free fuel economy. Further upgradation can also be done by coupling these reactions with organic carbon compounds, which will essentially form higher value‐added products like urea and different organonitrogen compounds using microrobots as the catalytic platform.

## 4 Experimental Section

### Materials

Bismuth(III) nitrate pentahydrate (Bi(NO_3_)_3_. 5H_2_O), potassium iodide (KI), nitric acid (HNO_3_), phosphotungstic acid hydrate (H_3_[PW_12_O_40_]. xH_2_O) (PTA), hydrochloric acid (HCl), copper(II) chloride dihydrate (CuCl_2_. 2H_2_O), iron(III) chloride hexahydrate (FeCl_3_·6H_2_O), sodium acetate (CH_3_COONa), polyethylenimine (PEI), ammonium hydroxide solution (NH_4_OH, 28% in H_2_O), ammonium chloride (NH_4_Cl), potassium hydroxide pellets (KOH), ethanol (C_2_H_5_OH, absolute), ethylene glycol (C_2_H_4_(OH)_2_), isopropanol (C_3_H_7_OH, HPLC grade), phenol (C_6_H_5_OH), formic acid (HCOOH), and benzyl alcohol (C_6_H_5_CH_2_OH) were purchased from Sigma‐Aldrich (Merck, Germany).

### Synthesis of BiOI

BiOI was synthesized according to previous reports.^[^ ^43^ ^]^ Typically, 200 mg of Bi(NO_3_)_3_. 5H_2_O was dissolved in an acidic aqueous solution of 5.5 mL containing 1 mL of 1 m HNO_3_ under stirring until a clear solution was obtained (solution A). Simultaneously, 70 mg of KI was dissolved in 11 mL of deionized (DI) water (solution B). Solution B was added dropwise to solution A under stirring. The mixture had a pH of ≈2. The pH of this mixture was adjusted to 7 using 1.5 m NH_4_OH. The mixture was stirred at 80 °C for 3 h at 600 rpm. A reddish solid precipitate was obtained which was collected by centrifugation followed by washing with DI water and ethanol and drying at 60 °C in an oven overnight.

### Fabrication of BiOI/PTA/Cu Microparticles

As‐synthesized BiOI (10 mmol) was dispersed in 10 mL of DI water containing 5 mmol of phosphotungstic acid (PTA) and was kept under magnetic stirring (300 rpm) for 12 h. The solid BiOI/PTA was isolated by centrifugation, followed by washing with DI water and drying in a vacuum. Four milliliters of BiOI/PTA dispersion (4 mg mL^−1^) were mixed with 1 mL of isopropanol, sealed, and N_2_ gas was purged into the dispersion for 30 min. The dispersion was irradiated with UV light using a customized 24 LED setup with twelve LZ1‐10UB0R LEDs and twelve LZ4‐20MA00‐0000 LEDs, maximum emission at 395 nm, 350 mA, power density 6 mW cm^−2^ for 3 h, which reduced W^6+^ centers of the BiOI bound PTA to W^5+^. After switching off the light source, 5 mL of 1 mm CuCl_2_.2H_2_O was added to the dispersion and was left to mature for 12 h. The reduced W^5+^ centers were reversibly oxidized back to W^6+^ and the Cu^2+^ centers were simultaneously reduced to Cu^0^/Cu^+^. The resulting BiOI/PTA/Cu products were isolated by centrifugation, followed by thorough washing with DI water and subsequently drying under vacuum.

### Fabrication of the AmmoGen Microrobots

Polyethylenimine (PEI)‐functionalized Fe_3_O_4_ (Fe_3_O_4_@PEI) NPs were synthesized by a solvothermal method.^[^ ^49^, ^72^ ^]^ Briefly, FeCl_3_·6H_2_O (1 g), sodium acetate (2.4 g), and PEI (2.0 g) were dissolved in 50 mL of ethylene glycol and mechanically stirred at 60 °C for 30 min. The resulting mixture was transferred to a Teflon‐lined stainless‐steel autoclave and heated at 200 °C for 10 h. The black products were washed thrice with ethanol and DI water and dried at 60 °C for 6 h. Typically, to prepare the AmmoGen microrobots, 5 mL of BiOI/PTA/Cu particles (10 mg mL^−1^) were mixed with 5 mL of Fe_3_O_4_@PEI NPs (1 mg mL^−1^) in an aqueous state. This mixture was mechanically stirred at 800 rpm for 30 min at room temperature using a vibratory shaker to form the hybrid structures.

### Morphological and Physicochemical Characterizations of the AmmGen Microrobots

The morphology and elemental composition of the BiOI/PTA/Cu, Fe_3_O_4_@PEI, and the AmmoGen microrobots were characterized using a JEOL JSM‐7610Fplus SEM equipped with an EDS detector. The light absorption spectra of the materials were recorded by a JASCO V‐730 double‐beam UV–vis spectrophotometer. Measurements of surface potentials were obtained using Malvern Zetasizer Ultra zeta potential analyzer in a DTS1070 folded capillary cell, controlled by the ZS XPLORER program. The crystallinity present in the samples was determined using an automated multipurpose XRD SmartLab (RIGAKU, Japan). FT‐IR spectra for the samples were measured using a Thermo Scientific Nicolet iS10 FT‐IR spectrometer. The XPS measurements were performed using the Nexsa G2 XPS system (Thermo Fisher Scientific) with a monochromatic source (Al‐Kα) and a photon energy of 1486.7 eV. All the spectra were measured in the vacuum of 2 × 10^−7 ^Pa and at a temperature of 20 °C. The survey spectra were measured with a pass energy of 150.00 eV and a step of 1.0 eV while for the high‐resolution spectra, a pass energy of 30.00 eV and an electronvolt step of 0.1 eV were used. Charge compensation was used for all measurements. The spectra were evaluated with the CasaXPS version 2.3.26PR 1.0 software.^[^ ^73^ ^]^

### Motion of the AmmoGen Microrobots in the Presence of a Rotating Magnetic Field

The motion of the AmmoGen microrobots was manipulated using a customized electromagnetic setup consisting of three pairs of orthogonal coils with built‐in functions for tuning frequencies and rotation angles of the transversal rotating magnetic field.^[^ ^31^, ^56^ ^]^ Throughout all the experiments the magnetic field was maintained at 5 mT. The motion generated by the rotating magnetic field was monitored and recorded using an inverted optical microscope. Typically, for a video recording, 30 µL of the dispersion containing microrobots (concentration 0.1 mg mL^−1^) was taken on a glass slide and then introduced to the magnetic field inside the coils. The microrobots in the video were analyzed using NIS‐Elements AR 3.2 software to obtain the average velocities and trajectories.

### Photosynthesis of Ammonia from Nitrates using AmmoGen Microrobots Under a Rotating Magnetic Field

For synthesizing ammonia, the microrobots were dispersed in an aqueous solution containing nitrates under ultrasonication. The dispersion was sealed and purged with Ar gas for 30 minutes. Then the dispersion was introduced inside the six‐coil electromagnetic setup described above, where the magnetic field was maintained at 5 mT and the frequencies used were 0 Hz (“static”), 1 Hz, and 5 Hz. The maximum yield was obtained for 5 Hz, so this frequency was maintained throughout all experiments. The motion of the microrobots was controlled using external random programmed motion at a frequency of 5 Hz. The “dynamic” microrobots were irradiated for 1 h using a customized LED 24‐lamp setup (twelve LZ1‐10UB0R‐00U6 LEDs and twelve LZ4‐20MA00‐0000 LEDs, 350 mA, power density 6 mW cm^−2^) mounted above magnetic coil at 20 cm from the sample stage. For optimizing the nitrate to ammonia reduction reaction, different reaction conditions were used‐ light sources used‐ full spectrum (300 W Xe lamp), green light (*λ*_max_ = 523 nm), and blue LED (*λ*_max_ = 460 nm); sacrificial agents used‐ ethylene glycol, formic acid, phenol, and benzyl alcohol. Reactions with similar reaction conditions and setup were also performed for “static” AmmoGen particles in the absence of the magnetic field. The microrobots were separated from the reaction mixture by using a neodymium magnet. The ammonia yield was measured using UV–vis spectroscopy with the help of a photometric ammonium detection kit (Spectroquant) using the indophenol blue method. For the reaction solutions with pH 2, the pH was adjusted to 4 using 1 m KOH solution, before performing the indophenol tests.

## Conflict of Interest

The authors declare no conflict of interest.

## Author Contributions

M.P., A.M., and J.K. conceived and designed the project and the experiments. A.M. and J.K. synthesized the materials and studied the magnetic motion of the microrobots. A.M. characterized and analyzed the data. A.M. carried out the photosynthesis of ammonia experiments. M.P., A.M., and J.K. wrote the manuscript. M.P. supervised the entire project. All the authors contributed to the discussion and manuscript preparation.

## Supporting information



Supporting Information

Supplemental Movie 1

Supplemental Movie 2

Supplemental Movie 3

Supplemental Movie 4

Supplemental Movie 5

Supplemental Movie 6

## Data Availability

The data that support the findings of this study are available from the corresponding author upon reasonable request.
