# Supplementary material for: Magnetically Propelled Microrobots toward Photosynthesis of Green Ammonia from Nitrates
Source: Small. 2024 Nov 11;21(14):2407050. doi: 10.1002/smll.202407050 (PMC11983241; doi:10.1002/smll.202407050)
Supplement: Supplementary file 1 — Supporting Information [file SMLL-21-2407050-s007.docx]

Supplementary Information

Magnetically Propelled Microrobots toward Photosynthesis of Green Ammonia from Nitrates

Apabrita Mallick^1^, Jeonghyo Kim^1^, Martin Pumera^1, 2^ *^*^*

^1^Advanced Nanorobots & Multiscale Robotics Laboratory, Faculty of Electrical Engineering and Computer Science, VSB − Technical University of Ostrava, 17. listopadu 2172/15, 708 00, Ostrava, Czech Republic

^2^Department of Medical Research, China Medical University Hospital, China Medical University, No. 91 Hsueh-Shih Road, Taichung 4040, Taiwan


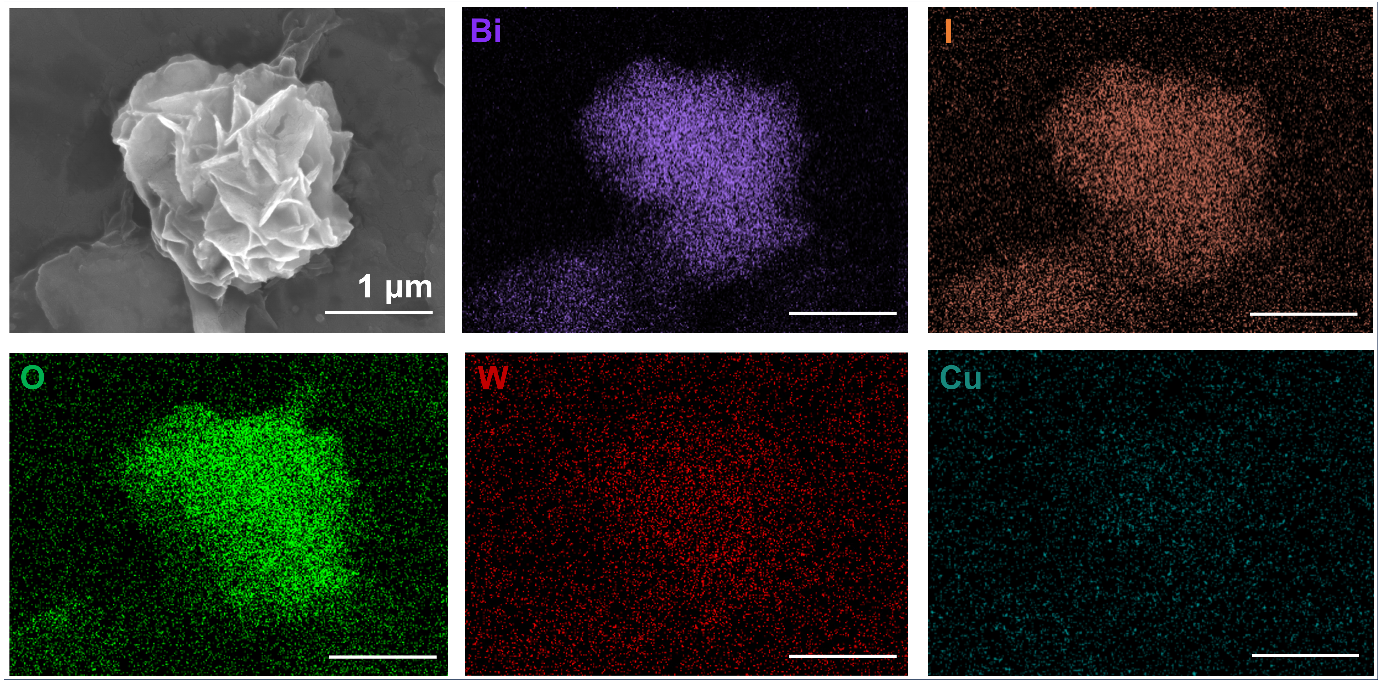


**Supplementary Figure S1.** Scanning electron microscopy and elemental dispersive X-ray analysis (SEM-EDAX) mapping of BiOI/PTA/Cu particles demonstrate the distribution of the microparticle's Bi, I, O, W, and Cu elements.


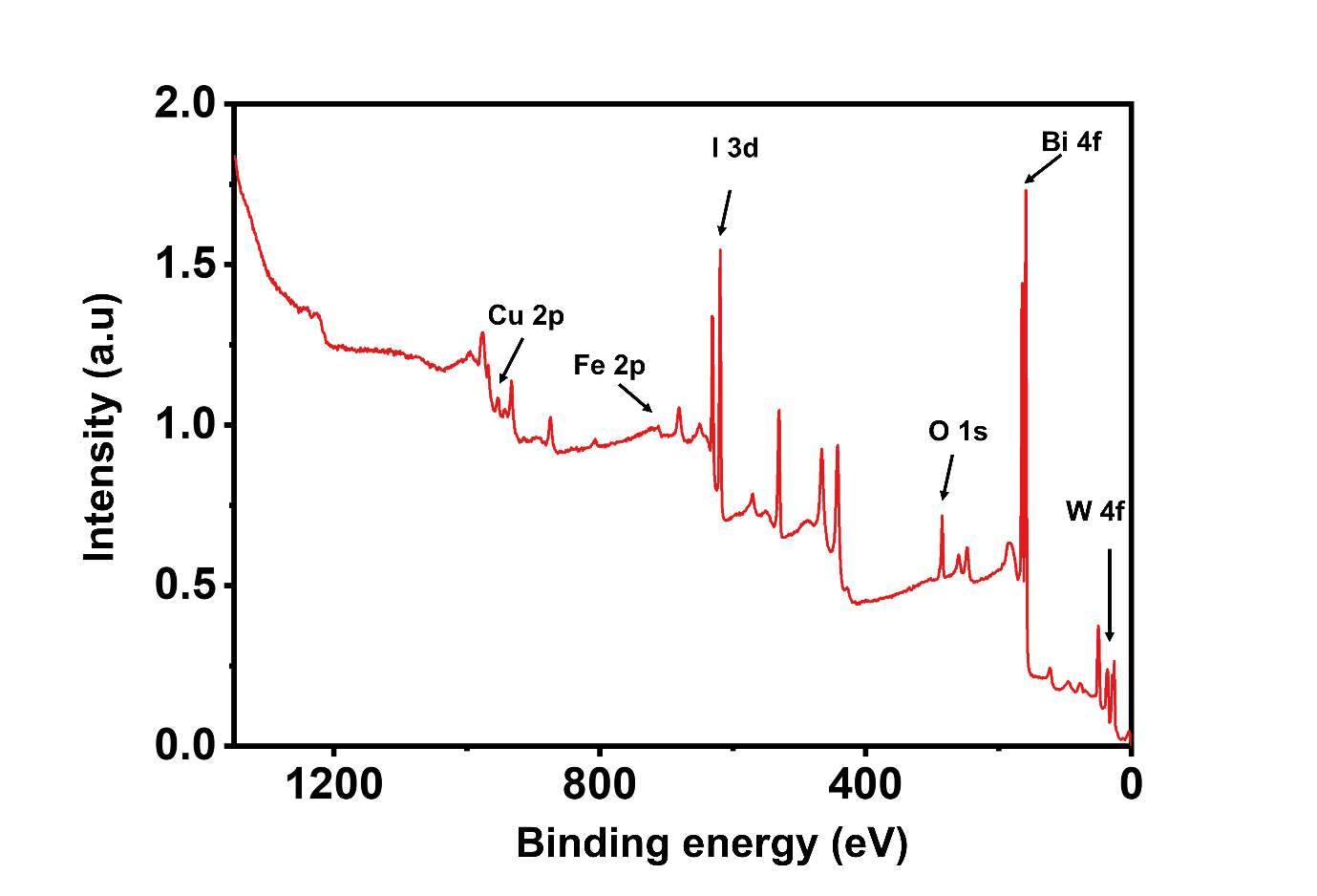


**Supplementary Figure S2.** X-ray photoelectron spectroscopy (XPS) survey spectra of the AmmoGen microrobots.

**Supplementary Table S1.** Results of deconvolution and peak fitting of the X-ray photoelectron spectroscopy (XPS) high-resolution spectra for the AmmoGen microrobots.

| **Core level** | **Peak position of Binding Energy (eV)** | **Assignment** | **Reference** |
| --- | --- | --- | --- |
| **Bi 4f** | 158.6 | Bi 4f_7/2_ (Bi^3+^) | 1 |
|  | 163.9 | Bi 4f_5/2_ (Bi^3+^) |  |
| **I 3d** | 618.5 | I 3d_5/2_ (I^-^) | 1 |
|  | 630.0 | I 3d_3/2_ (I^-^) |  |
| **W 4f** | 35.0 | W 4f_7/2_ (W^6+^) | 2 |
|  | 37.1 | W 4f_5/2_ (W^6+^) |  |
| **Cu 2p** | 931.9 | Cu 2p_3/2_ (Cu^+^/Cu^0^) | 3 |
|  | 951.8 | Cu 2p_1/2_ (Cu^+^/Cu^0^) |  |
|  | 933.4 | Cu 2p_3/2_ (Cu^2+^) |  |
|  | 952.8 | Cu 2p_1/2_ (Cu^2+^) |  |
|  | 929.9 | Satellite |  |
|  | 943.1 | Satellite |  |
| **Fe 2p** | 710.3 | Fe 2p_3/2_ (Fe^2+^) | 4 |
|  | 723.6 | Fe 2p_1/2_ (Fe^2+^) |  |
|  | 712.7 | Fe 2p_3/2_ (Fe^3+^) |  |
|  | 726.5 | Fe 2p_1/2_ (Fe^3+^) |  |
|  | 718.2 | Satellite |  |
|  | 732.4 | Satellite |  |
| **O 1s** | 529.7 | O^2-^ | 5 |
|  | 531.2 | O_ads_ |  |


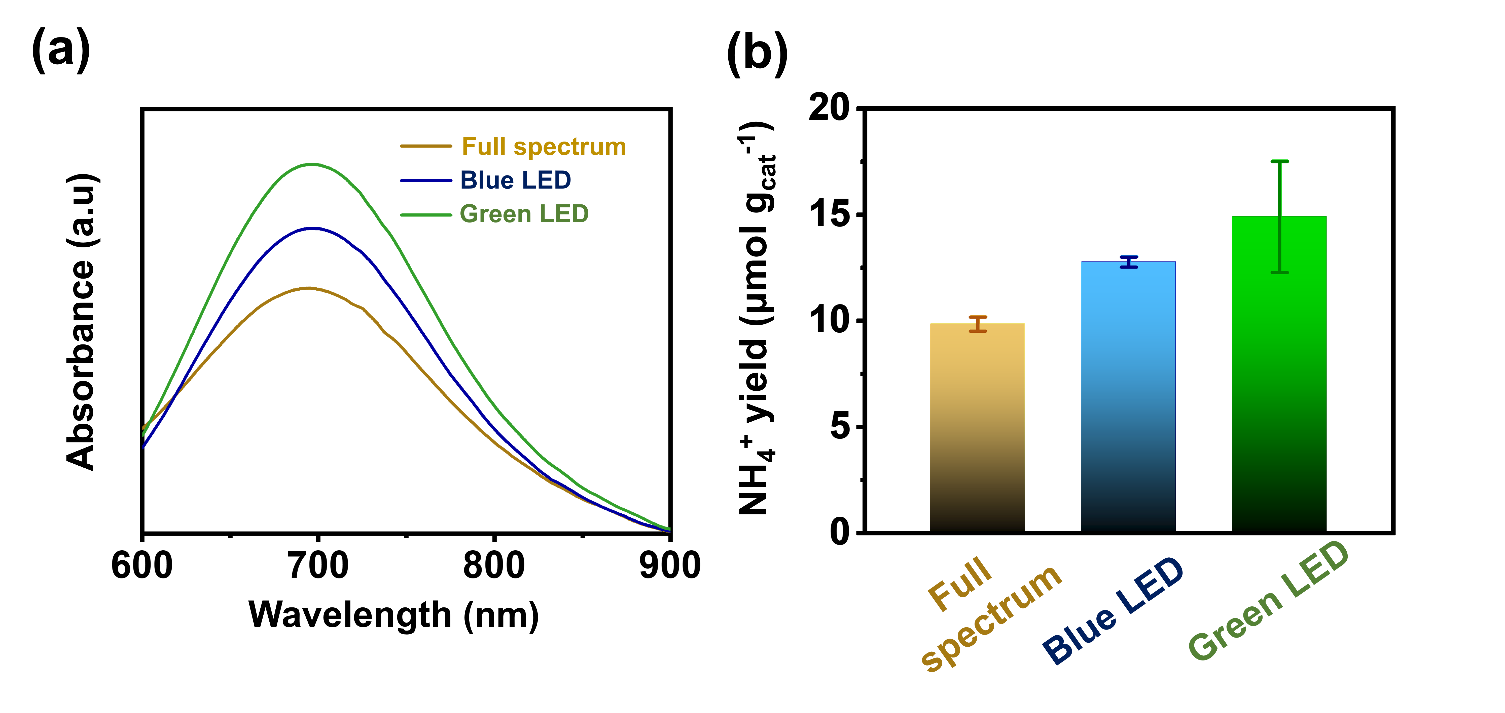


**Supplementary Figure S3. Optimization of the light source. (**a) UV-visible absorption spectra obtained after reaction with irradiation of different light sources, i.e.; full spectrum, blue LED, and green LED using ‘static’ Ammogen particles; (b) Yield of [NH_4_^+^] obtained by colorimetric indophenol method for three different light sources, i.e.; full spectrum, blue LED, and green LED using ‘static’ Ammogen particles.


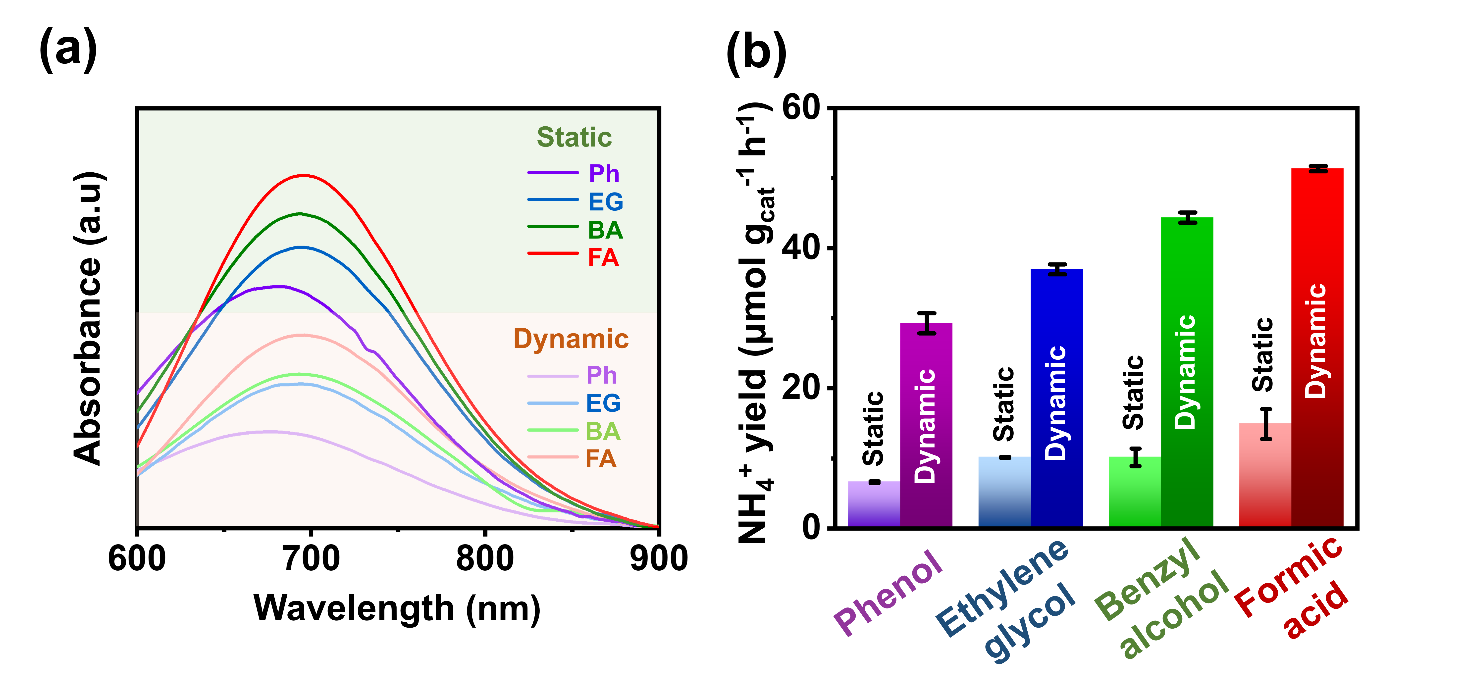


**Supplementary Figure S4. Optimization of the sacrificial agent. (**a) UV-visible absorption spectra obtained after reaction with different sacrificial agents, i.e.; phenol (Ph), ethylene glycol (EG), benzyl alcohol (BA), and formic acid (FA) using ‘static’ and ‘dynamic’ Ammogen microrobots. All the spectra have been calibrated with respect to blank experiments with the respective sacrificial agents. The presence of phenol induces a blueshift of λ_max_ by 12 nm; (b) Yields of [NH_4_^+^] obtained by colorimetric indophenol method for different sacrificial agents, i.e.; phenol, ethylene glycol, benzyl alcohol, and formic acid using ‘static’ and ‘dynamic’ Ammogen microrobots.


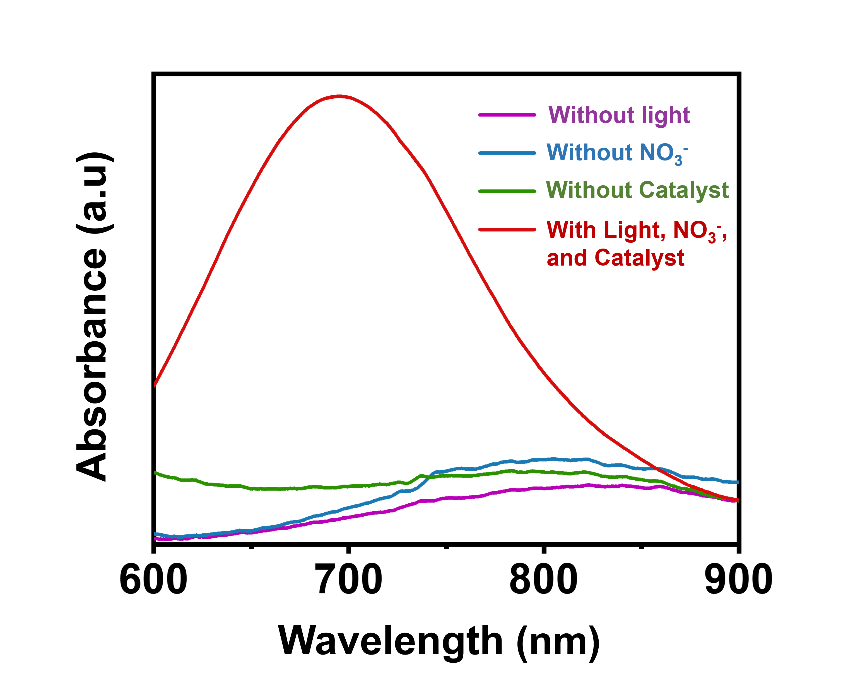


**Supplementary Figure S5. Control experiments.** UV-visible absorption spectra were obtained after the reaction carried out (i) without green LED light source, (ii) without starting substrate, NO_3_^-^, (iii) without the AmmoGen particles, and compared with (iv) reaction carried out with all three components.


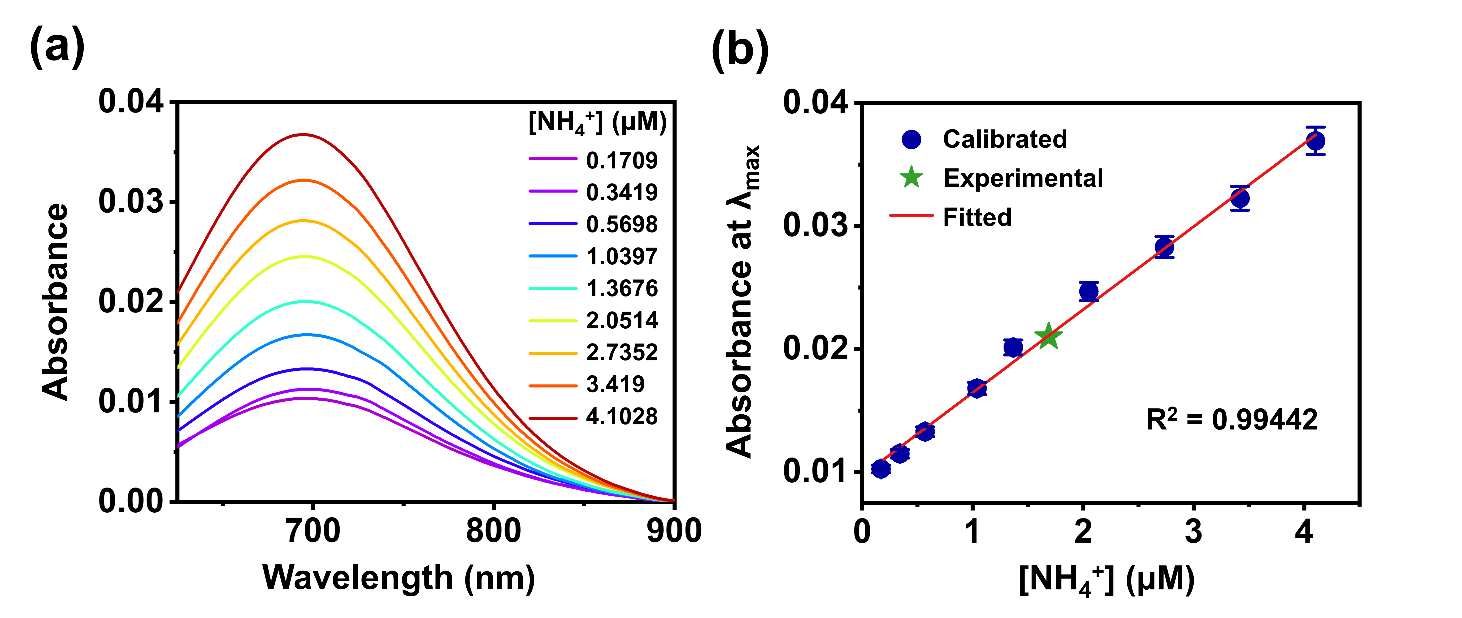


**Supplementary Figure S6. Calibration experiments. (**a) UV-visible absorption spectra of solutions containing the calculated amounts of NH_4_Cl, (b) calibration curve for determination of [NH_4_^+^] by colorimetric indophenol method.


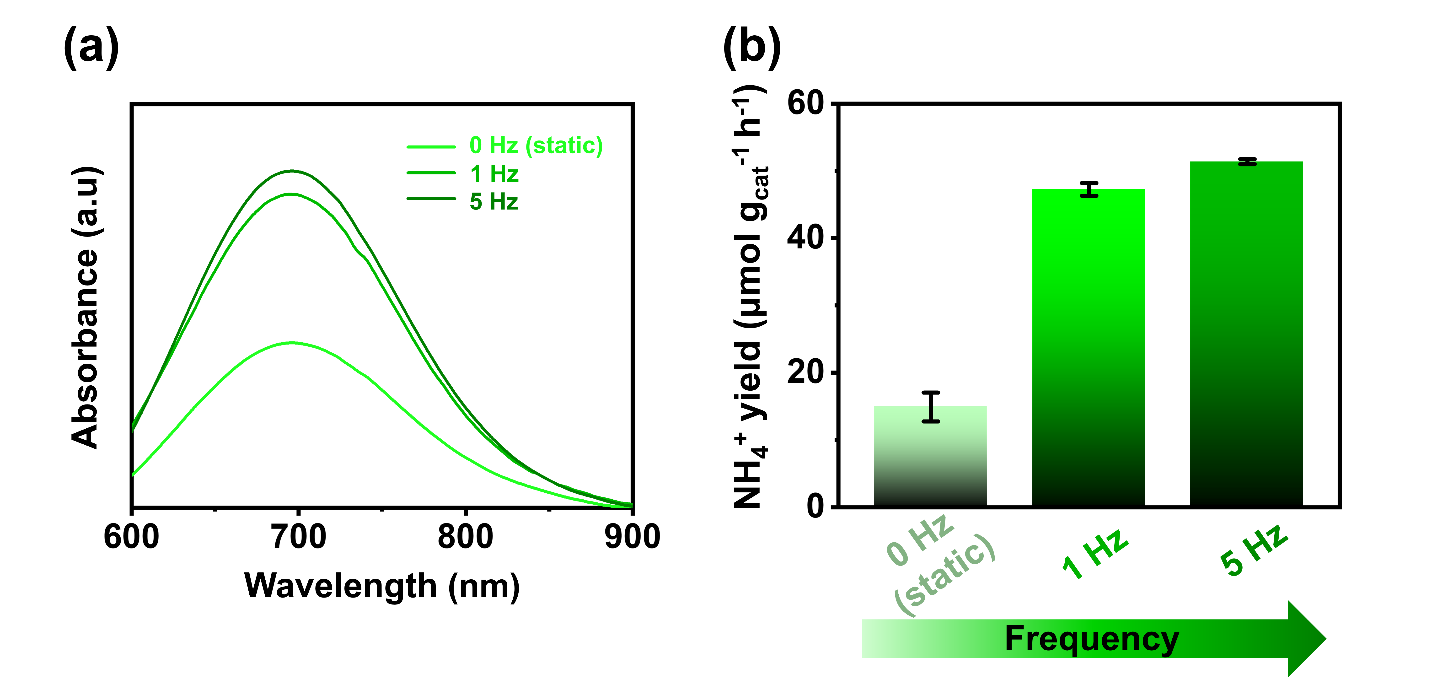


**Supplementary Figure S7: Photocatalytic reactions under different magnetic fields. (**a) UV-visible absorption spectra obtained after reactions at different frequencies of the magnetic fields, i.e.; 0 Hz (static particles), 1 Hz, and 5 Hz; (b) Yields of [NH_4_^+^] obtained by colorimetric indophenol method for different frequencies of the magnetic fields, i.e.; 0 Hz (static particles), 1 Hz, and 5 Hz.

**References:**

1. Mayorga-Burrezo, P.; Mayorga-Martinez, C. C.; Kim, J.; Pumera, M., Hybrid magneto-photocatalytic microrobots for sunscreens pollutants decontamination. *Chem. Eng. J.* **2022,** *446*, 137139.

2. Tan, H.; Rong, S.; Zong, Z.; Zhang, P.; Zhao, R.; Song, F.; Cui, H.; Chen, Z.-N.; Yi, W.; Zhang, F., Surface-sealing encapsulation of phosphotungstic acid in microporous UiO-66 as a bifunctional catalyst for transfer hydrogenation of levulinic acid to γ-valerolactone. *Phys. Chem. Chem. Phys.* **2023,** *25* (27), 18215-18223.

3. Sahai, A.; Goswami, N.; Kaushik, S.; Tripathi, S., Cu/Cu2O/CuO nanoparticles: Novel synthesis by exploding wire technique and extensive characterization. *Appl. Surf. Sci.* **2016,** *390*, 974-983.

4. Wilson, D.; Langell, M., XPS analysis of oleylamine/oleic acid capped Fe_3_O_4_ nanoparticles as a function of temperature. *Appl. Surf. Sci.* **2014,** *303*, 6-13.

5. Fan, K.; Zou, H.; Duan, L.; Sun, L., Selectively etching vanadium oxide to modulate surface vacancies of unary metal–based electrocatalysts for high‐performance water oxidation. *Adv. Energy Mater.* **2020,** *10* (5), 1903571.
